# Testing and correcting for weak and pleiotropic instruments in two‐sample multivariable Mendelian randomization

**DOI:** 10.1002/sim.9133

**Published:** 2021-08-02

**Authors:** Eleanor Sanderson, Wes Spiller, Jack Bowden

**Affiliations:** ^1^ MRC Integrative Epidemiology Unit University of Bristol Bristol UK; ^2^ Population Health Sciences University of Bristol Bristol UK; ^3^ College of Medicine and Health University of Exeter Exeter UK

**Keywords:** Cochran's *Q*‐statistic, instrument strength, instrument validity, multivariable Mendelian randomization, two‐sample Mendelian randomization

## Abstract

Multivariable Mendelian randomization (MVMR) is a form of instrumental variable analysis which estimates the direct effect of multiple exposures on an outcome using genetic variants as instruments. Mendelian randomization and MVMR are frequently conducted using two‐sample summary data where the association of the genetic variants with the exposures and outcome are obtained from separate samples. If the genetic variants are only weakly associated with the exposures either individually or conditionally, given the other exposures in the model, then standard inverse variance weighting will yield biased estimates for the effect of each exposure. Here, we develop a two‐sample conditional *F*‐statistic to test whether the genetic variants strongly predict each exposure conditional on the other exposures included in a MVMR model. We show formally that this test is equivalent to the individual level data conditional *F*‐statistic, indicating that conventional rule‐of‐thumb critical values of F> 10, can be used to test for weak instruments. We then demonstrate how reliable estimates of the causal effect of each exposure on the outcome can be obtained in the presence of weak instruments and pleiotropy, by repurposing a commonly used heterogeneity *Q*‐statistic as an estimating equation. Furthermore, the minimized value of this *Q*‐statistic yields an exact test for heterogeneity due to pleiotropy. We illustrate our methods with an application to estimate the causal effect of blood lipid fractions on age‐related macular degeneration.

## 1 INTRODUCTION

Instrumental variables (IV) is a form of regression analysis which estimates the causal effect of an exposure on an outcome in the presence of unobserved confounding. Mendelian randomization (MR) is a rapidly expanding application of the IV method in the field of epidemiology in which genetic variants are used as instruments. If genetic variants—usually single nucleotide polymorphisms (SNPs)—are available which reliably predict the exposure and are not associated with the outcome through any other pathway, then they are valid IVs. These genetic variants can then be used as instruments to obtain an estimate for the causal effect of a modifiable health exposure on a disease outcome.^1^, ^2^ The results of such an analysis can inform the development of public health, or even pharmaceutical, interventions. MR is often conducted with summary‐level data on the SNP‐exposure and SNP‐outcome associations obtained from genome‐wide association studies (GWAS) without the need to have individual level data on the genetic variants, exposure and outcome available to the researcher conducting the MR study.

Multivariable Mendelian randomization (MVMR) is a recently developed extension of MR that can be applied with either individual or summary level data to estimate the effect of multiple, potentially related, exposures on an outcome.^3^, ^4^ The three core assumptions that define a set of SNPs, *G*, as valid IV's for the purpose of an MVMR analysis are;

IV1: *G* must be strongly associated with *each* exposure given the other exposures included in the model;
IV2: *G* is independent of all confounders of *any* of the exposures and the outcome; and
IV3: *G* is independent of the outcome given *all* of the exposures.^4^

**FIGURE 1:**
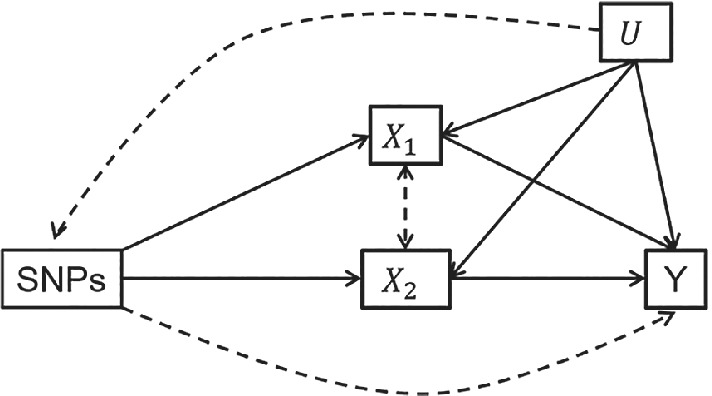
Assumptions for a MVMR analysis: DAG illustrating the assumptions required for MVMR. Dashed lines represent associations that must not exist for the SNPs to be valid instruments for the set of exposures. DAG, directed acyclic graph; MVMR, multivariable Mendelian randomization; SNP, single nucleotide polymorphism

These assumptions are shown in Figure 1. A violation of IV1 induces ‘weak instrument bias’ in the resulting estimates.^5^, ^6^ In a conventional (univariable) MR analysis, the definition of instrument strength is straightforward and unambiguous. Assumption IV1 can be tested with an *F*‐statistic, which tests the association between the SNP and the exposure. When univariable MR analysis based on individual level data from a single sample, if the *F*‐statistic is larger than the rule‐of‐thumb value of 10 then the SNPs are said to be a “strong” instrument. We can then reject the null hypothesis that the instruments are weak in the sense that the bias of the MR estimate is equal to or greater than 10% of the observational (or ordinary least squares, OLS) association.^5^, ^6^ In any MVMR analysis it is necessary that there are at least as many instruments as exposures and that this *F*‐statistic is large for each exposure included, however this is no longer sufficient; the SNP's used as IV's also need to predict each exposure conditional on the other predicted exposures included in the estimation. This additional condition ensures that there is sufficient variation in association between the SNPs and each exposure, to avoid a problem of weak instrument bias in the MVMR model. Unlike in univariable MR, in MVMR weak instrument bias can bias the estimated effect of each exposure either towards or away from the null. This makes testing for weak instruments in any MVMR estimation particularly important.

With individual level data, weak instruments can be tested in MVMR using the Sanderson‐Windmeijer conditional *F*‐statistic, denoted $F_{\text{SW}}$.^4^, ^7^ Under weak instruments $F_{\text{SW}}$ has the same distribution as the conventional *F*‐statistic and so can be compared with the same critical values.^5^, ^6^ Therefore, when testing for weak instruments, verifying that $F_{\text{SW}}$ is greater than the rule‐of‐thumb of 10 means that we can reject the null hypothesis that the average bias of the MVMR estimates is at least 10% of the bias of the equivalent multivariable OLS estimates.

When individual level data on the genetic variants, exposure and outcome are not available two‐sample MVMR can be conducted using summary data estimates of SNP‐exposure and SNP‐outcome associations. In two‐sample MR, weak instruments bias the causal estimates towards the null rather than the observational association.^8^ In this article, we consider testing for weak instruments and estimation in the presence of weak instruments in the summary‐data MVMR setting. Sanderson et al^4^ derived a *Q* statistic ($Q_{x_{j}}$) to test for underidentification (ie, where the SNPs explain none of the variation in an exposure) in two‐sample MVMR. We formally show in this article that a transformation of this statistic has the same distribution as $F_{\text{SW}}$ and therefore can also be compared with standard weak instrument critical values, or rule‐of‐thumb of F>10, to test for weak instruments in the two sample setting.

We then go on to consider horizontal pleiotropy in MVMR. Horizontal pleiotropy is a major threat to the validity of an MR analysis. It occurs when the SNPs have an effect on the outcome (either directly or through another exposure not included in the model) that is not via the exposure of interest, as illustrated by the dashed arrow from *G* to *Y* in Figure 1. This violates assumption IV3 and can lead to biased estimates of the causal effect of each exposure on the outcome from an MR analysis.^9^ Horizontal pleiotropy can be either “balanced,” where the pleiotropic effects of the SNPs in the estimation are evenly distributed between having positive and negative effects on the outcome and so have no overall directional effect, or “unbalanced” where on average these pleiotropic effects act in one direction on the outcome. IVW estimation and MVMR‐IVW estimation are robust to balanced pleiotropy when the instruments are strong. However, this no longer holds if the exposures are only weakly predicted by the SNPs. A number of methods currently exist for univariable MR estimation that are robust to pleiotropy under different assumptions.^10^, ^11^, ^12^, ^13^ MVMR can mitigate horizontal pleiotropy via known pleiotropic pathways through the inclusion of multiple exposures, however limited methods are available for pleiotropy robust MVMR models.^4^, ^14^, ^15^ Furthermore, in the presence of weak instruments standard tests are increasingly likely to detect pleiotropy when in truth none is present. The major contribution of this article is to extend weak instrument and pleiotropy robust estimation to two sample MVMR with an arbitrary number of exposures. Furthermore, we show that a heterogeneity statistic derived within this estimation procedure provides an exact test for the presence of pleiotropy in the presence of weak instruments. The methods presented here therefore provide the statistical framework for accurate and reliable MVMR model fitting, with potentially large numbers of exposures, in the presence of weak instruments and pleiotropy.

We apply our methods to determine whether particular subsets of metabolites can be strongly predicted by 150 SNPs associated with at least one of 118 metabolites using data first presented by Kettunen et al^16^ and estimate the causal effect of those traits on age‐related macular degeneration (AMD). The two‐sample conditional *F*‐statistic calculated for these data highlights that it is not possible to strongly predict multiple metabolites from the same subgroup despite each lipid fraction having a moderately high individual *F*‐statistic and that any MVMR estimates including these is likely to be biased. Any analyst naively applying MVMR methods to such data without the correct diagnostic statistics to hand is in danger of generating poor quality results.

Finally, we present an R package (“MVMR”) that can conduct MVMR‐IVW estimation and calculate all of the test statistics and estimators discussed in this article.

## 2 A TEST FOR WEAK INSTRUMENTS

Let $X=(X_{1},X_{2},\ldots ,X_{K})$ be a set of *K* exposure variables and let *G* be a set of *L* instruments $G=(G_{1},G_{2},\ldots ,G_{L})$. Define the K×L matrix of associations between each exposure and each instrument as;

(1) $$\Pi =\left(\begin{array}{cccc} \pi_{11} & \pi_{12} & \ldots & \pi_{1L}\\ \pi_{21} & \pi_{22} & \ldots & \pi_{2L}\\ \vdots & \vdots & \ddots & \vdots \\ \pi_{K1} & \pi_{K2} & \ldots & \pi_{KL} \end{array}\right),$$

where for example $\pi_{32}$ represents the association between exposure 3 and SNP 2. Without loss of generality, testing whether the instrument set *G* can explain variation in a single exposure, $X_{1}$, conditional on all other exposures $\left(X_{2},\ldots ,X_{K}\right)$ is equivalent to testing whether model (2) below is identified

(2) $$\begin{array}{ll} X_{1} & =\delta_{01}+\delta_{1}X_{-1}+\epsilon_{1} \end{array}$$

(3) $$\begin{array}{ll} X_{m} & =\pi_{0m}+\overset{L}{\underset{j=1}{\sum }}\pi_{mj}G_{j}+\epsilon_{m},\;m=2,\ldots ,K \end{array}$$

Here: $\delta_{01}$ and each $\pi_{0m}$ are scalar parameters; $\delta_{1}$ is a K−1 vector of parameters, and $\epsilon_{1}$ and $\epsilon_{m}$ are random error terms. Collecting $\pi_{2},\ldots ,\pi_{K}$ into a single (K−1)×L matrix, define $\Pi_{-1}$ as the matrix Π minus its first row. This model considers only the exposures, and not the outcome, of the main estimation of interest as we wish to test whether the instruments explain any variation in $X_{1}$ over and above the variation explained in all of the other exposures. If this model is overidentified then the rank of $\Pi_{-1}$ is strictly greater than K−(L−1) and the instruments can strongly predict $X_{1}$ conditional on all other exposures included in the estimation.

In two sample summary data settings we do not directly observe exposures $X_{1},\ldots ,X_{K}$, only estimates for the K×L SNP‐exposure associations that define $\hat{\Pi }$ the estimated value of Π obtained through regression of each exposure on each SNP. However, we can use these association estimates to define an analogous formula to (2)

$${\hat{\pi }}_{1}=\delta_{1}{\hat{\Pi }}_{-1}+v_{1}$$

The *Q* statistic for exposure 1 based on the summary data estimates can be written as;

(4) $$Q_{x_{1}}=\overset{L}{\underset{j=1}{\sum }}\left(\frac{1}{\sigma_{x_{1j}}^{2}}\right){\left({\hat{\pi }}_{1j}-{\tilde{\delta }}_{1}{\hat{\Pi }}_{-1j}\right)}^{2}$$

where the variance term $\sigma_{x_{1}j}^{2}$ is given by;

$$\sigma_{x_{1},j}^{2}={\tilde{\delta }}^{*}\sum_{V,j}{\left({\tilde{\delta }}^{*}\right)}^{\prime }$$

Where *j* represents an individual SNP and $\pi_{1j}$ and $\Pi_{-1j}$ represent column *j* of $\pi_{1}$ and $\Pi_{-1}$, respectively. ${\tilde{\delta }}^{*}$ is the K by 1 vector $\left(-1\;\;{\tilde{\delta }}_{2}\;\;\ldots \;\;{\hat{\delta }}_{K}\right)$, and ${\tilde{\delta }}_{k}$ is a consistent estimator for $\delta_{k}$, for example estimated through an inverse variance weighted (IVW) least squares regression of ${\hat{\pi }}_{1}$ on ${\hat{\Pi }}_{-1}$. The matrix $\sum_{V,j}$ defines the covariance of the estimated effects of snp j on each of the exposures:

(5) $$\sum_{V,j}=\left(\begin{array}{cccc} \sigma_{1,j}^{2} & \sigma_{12,j} & \ldots & \sigma_{1K,j}\\ \sigma_{12,j} & \sigma_{2,j}^{2} & \ldots & \sigma_{2K,j}\\ \vdots & \vdots & \ddots & \vdots \\ \sigma_{1K,j} & \sigma_{2K,j} & \ldots & \sigma_{K,j}^{2} \end{array}\right)$$

If each ${\hat{\pi }}_{kj}$ is obtained separately via univariable regressions with an intercept, then the error terms are obtained from the expressions:

(6) $$\sigma_{k,j}^{2}=\frac{{\left(G_{j}^{T}G_{j}\right)}^{-1}}{n}\overset{n}{\underset{i=1}{\sum }}{\hat{v}}_{ki}^{2},\;\text{and}\;\sigma_{km,j}=\frac{{\left(G_{j}^{T}G_{j}\right)}^{-1}}{n}\overset{n}{\underset{i=1}{\sum }}{\hat{v}}_{ki}{\hat{v}}_{mi},\;k\neq m$$

Where $v_{k,i}$ and $v_{m,i}$ are the residual error terms for univariable regressions of SNP *i* on exposures *k* and *m*, respectively. Under the null hypothesis that the instruments do not contain enough information to predict both exposure variables, $Q_{x_{1}}$ will be asymptotically $\chi_{L-1}^{2}$ distributed where *L* is the number of SNPs in the estimation. Rejecting the null hypothesis indicates that the SNPs can predict $X_{1}$ conditional on $X_{2}$. Dividing the *Q*‐statistic described above by the number of instruments, adjusted for the number of exposures, in the model gives a test statistic that is equivalent to the one sample conditional F statistic $F_{SW}$. Two‐sample MVMR‐IVW estimation is asymptotically equivalent to individual level two‐stage least squares estimation and therefore this test statistic can be applied to test for weak instrument in two‐sample MVMR in the same way as the conditional *F*‐statistic for individual level data.^17^

(7) $$\begin{array}{ll} F_{TS,k} & =\frac{Q_{x_{k}}}{L-(K-1)}\\ & ∼\frac{\chi_{\left(L-(K-1)\right)}^{2}}{L-(K-1)} \end{array}$$

Where $Q_{x_{k}}$ is the expression given in Equation (4).

### 2.1 Critical values

Comparing this statistic to standard critical values from the *F*‐distribution provides a test for a lack of identification. However, even if the genetic instruments explain some of the variation in the exposure they could still be “weak.” In this case the estimates obtained from the MVMR estimation could still be considerably biased. The one sample conditional *F*‐statistic ($F_{\text{SW}}$) has the same distribution as the Stock‐Yogo weak instrument test.^6^ Therefore, we can apply its weak instrument critical values to identify weak instrument bias for univariable and multivariable two‐sample MR.^5^, ^6^, ^7^ The weak instrument critical values derived by Stock and Yogo for the bias of the 2SLS estimator relative to the OLS estimator are derived under the definition that the instruments are weak when the bias of the IV estimator relative to the OLS estimator is at least 10%. The measure of relative bias used is the squared bias of the IV estimator ($\beta_{IV}$) relative to the squared bias of the OLS estimator ($\beta_{\text{OLS}}$). This is given by the equation;

$$B^{2}=\frac{{\left(E{\hat{\beta }}_{IV}-\beta \right)}^{\prime }\sum_{X}(E{\hat{\beta }}_{IV}-\beta )}{{\left(E{\hat{\beta }}_{\text{OLS}}-\beta \right)}^{\prime }\sum_{X}(E{\hat{\beta }}_{\text{OLS}}-\beta )}$$

Where $\sum_{X}=p\lim\frac{1}{n}X^{\prime }X$ and *X* here represents the n×K matrix of all of the exposures included in the estimation, *n* is the sample size. Calculating the bias in this way standardizes the exposures X so they are orthogonal and have unit SD. However, it means that the bias of the estimated effect of any particular exposure may differ from 10% and could act in the opposite direction to the bias of the model as a whole. If $F_{\text{TS}}$ is larger than the relevant Stock‐Yogo critical value we can reject the null hypothesis that the exposure is only weakly predicted by the instruments. These critical values have only been derived for models including up to 30 instruments, therefore in Table 1 we provide critical values for a larger range of instruments to test for a 5%, 10%, or 20% relative bias. These critical values are often approximated to a rule of thumb of F>10 to test a null hypothesis that the bias is at least 10% of the bias of the OLS estimator. The critical values given above also show that the rule of thumb of 10 is slightly smaller than the true critical value for this test and would lead to the null hypothesis being rejected more frequently. The two sample $F_{\text{TS}}$ statistic tests the bias of the model as a whole, this means that the sign of the bias of an individual causal parameter may differ from that of the model's bias, which is averaged across all of its constituent parameters. It also indicates that some weakly predicted exposures could be biased away from the null hypothesis.

**TABLE 1:** Critical values for conditional weak instrument tests

	Relative bias		
$k_{Z}$	5%	10%	20%
25	21.37	11.44	6.19
50	21.26	11.14	5.86
100	21.02	10.84	5.64
200	20.79	10.61	5.46
300	20.62	10.52	5.38
400	20.56	10.45	5.32
500	20.50	10.40	5.29

## 3 WEAK INSTRUMENT ROBUST TWO‐SAMPLE MVMR

### 3.1 Estimation in the presence of weak instruments

In the presence of weak instruments, standard IVW estimation of the MVMR mode, which we refer to as MVMR‐IVW, is biased. The LIML estimator has previously been proposed as an alternative estimator for individual‐level MR as it is less biased when there are many weak instruments.^18^ In the two‐sample summary data setting, Bowden et al^19^ and Zhao et al^20^ show that weak instruments can be effectively mitigated through minimization of an appropriate heterogeneity statistic using weights that account for the variance of the SNP‐exposure associations is analogs to one‐sample LIML estimation. It gives results that are substantially less biased than conventional regression based IVW estimates in the presence of a nonzero causal effect. The weak instrument robust estimation proposed by Bowden et al can be extended to the MVMR setting as a minimization of;

(8) $$Q_{A}=\overset{L}{\underset{j=1}{\sum }}\left(\frac{1}{\sigma_{A,j}^{2}}\right){\left({\hat{\Gamma }}_{j}-\beta^{\prime }{\hat{\pi }}_{j}\right)}^{2}$$

over β. Where β is a vector of causal parameters (to be estimated), ${\hat{\Gamma }}_{j}$ is the estimated effect of SNP *j* on the outcome, ${\hat{\pi }}_{j}$ is a vector of effects of SNP *j* on each exposure included in the estimation (ie, a column of the matrix Π) and;

(9) $$\sigma_{A,j}^{2}=\sigma_{y,j}^{2}+\beta^{\prime }\sum_{V,j}\beta .$$

Here, $\sigma_{y,j}^{2}$ is the variance of the estimated effect of the SNPs on the outcome, and $\sum_{V,j}$ is the variance‐covariance matrix defined in Equation (5). This is equivalent to minimization of the $Q_{A}$ statistic to test for heterogeneity described in Sanderson et al^4^ extended to a model with more than two exposures. We label estimates for β obtained in this manner as ${\hat{\beta }}_{Q}$. The standard MVMR‐IVW estimate is vunerable to weak instrument bias because instead of minimizing $Q_{A}$ in (8) using the full weights defined in (9) it incorrectly assumes that $\sigma_{A_{j}}^{2}=\sigma_{y_{j}}^{2}$. This ignores the component of variation from $\beta^{\prime }\sum_{j}\beta$ and is only valid if either all elements of β are zero or $\sum_{j}$ is negligible in comparison to $\sigma_{yj}^{2}$.

### 3.2 Testing for pleiotropy in the presence of weak instruments

Horizontal pleiotropy, where genetic variants influence the outcome through multiple phenotypes, can lead to a violation of the IV assumptions if they are not included as exposures in the MVMR estimation. Under the assumption that not all the SNPs included in the estimation have a pleiotropic effect on the outcome through the same pathway, this will lead to greater variation in the estimated causal effect of the exposures on the outcome than would be expected by chance. This excess heterogeneity can be reliably tested for using the minimized $Q_{A}$ statistic. More formally if all SNPs used in the MVMR analysis are valid instruments, in the sense that they identify a common set of causal parameters β, we would expect the $Q_{A}$ statistic in (6) evaluated at $\beta ={\hat{\beta }}_{Q}$ to follow a Chi‐squared distribution with L‐K degrees of freedom. Crucially, the test is exact in the sense that it will achieve its nominal type I error rate, even in the presence of weak instruments.^21^ The standard *Q*‐statistic used to generate the MVMR‐IVW estimate by setting $\sigma_{A,j}^{2}=\sigma_{y,j}^{2}$, referred to here as $Q_{\text{IVW}}$, will generally have an inflated type 1 error rate (ie, will detect pleiotropy too often when none is present) unless all $\beta^{\prime }\sum_{j}\beta$ terms are negligible.

### 3.3 Estimation with pleiotropic and weak instruments

Estimation of β through minimization of (8) will give estimates of the direct effect of each exposure on the outcome that are robust to weak instruments. However, these estimates will still be biased in the presence of pleiotropy. In order to account for heterogeneity due to pleiotropy, we extend the estimation of β by adding a pleiotropy variance parameter $\tau^{2}$ to the multivariable Q estimation and finding the joint value of $\left(\beta ,\tau^{2}\right)$ which minimizes;

$$\begin{array}{llll} & \overset{L}{\underset{j=1}{\sum }}\left(\frac{1}{\sigma_{A,j}^{2}}\right){\left({\hat{\Gamma }}_{j}-\beta^{\prime }{\hat{\pi }}_{j}\right)}^{2}-(L-K)=0\; & & \\ & \sigma_{A,j}^{2}=\sigma_{y,j}^{2}+\beta^{\prime }\sum_{j}\beta +\tau^{2}\; & & \end{array}$$

subject to;

$$\frac{\partial \sum_{j=1}^{L}\left(\frac{1}{\sigma_{A,j}^{2}}\right){\left({\hat{\Gamma }}_{j}-\beta^{\prime }{\hat{\pi }}_{j}\right)}^{2}}{\partial \beta }=0$$

We refer to the causal estimates derived in this way as ${\hat{\beta }}_{Q,\text{het}}$. This is an extension of the method described in Bowden et al for univariable MR to the MVMR setting.^19^ This method will account for balanced pleiotropy which biases the MVMR‐IVW estimates further in the presence of weak instruments by accounting for excess heterogeneity in the per SNP estimated effects that is not related to the variance in the SNP‐exposure associations or SNP‐outcome associations. It will not however account for directional pleiotropy where the pleiotropic effects of the SNPs on the outcome all, or mostly, act in one direction to either increase or decrease the outcome. However, it is possible to look at the individual contribution of each SNP to $Q_{A}$ to identify the largest outliers. If a small number of SNPs are observed to have a large effect on $Q_{A}$ they can potentially be removed as a sensitivity analysis and the MVMR model reestimated without them.

### 3.4 Confidence intervals for estimated effects

Estimation of β and $\tau^{2}$ through minimization of $Q_{A}$, does not provide readily available and reliable standard errors (SEs). We therefore suggest that SEs are obtained, and confidence intervals calculated, through a Jackknife procedure.

We propose the use of Jackknife rather than a bootstrap as with a moderate number of SNPs the repeated sampling in a bootstrap can lead to very weak instruments in any particular iteration even when the model has relatively strong instruments as a whole. A jackknife procedure estimates the model leaving out each SNP in turn and then calculates the SD of the effect estimate from these results. As each iteration includes all but one of the SNPs and includes each SNP only once this is unlikely to be affected by weak instruments due to the exclusion of some SNPs. When the number of SNPs used in the estimation is very small neither a Jackknife or bootstrap approach will calculate appropriate SEs however many applications of MVMR include 100 to 200 SNPs as instruments and with this number of SNPs a jackknife approach will be feasible.

## 4 ESTIMATION OF $\sum_{Vj}$

So far we have assumed that the pairwise covariance between a set of SNP's estimated association with any two exposures is known for all exposures and all SNPs. However, this data is not generally reported by GWAS summary statistics. Similarly, it would not be feasible for these studies to report this data due to the large number of potential covariances that could be required for all potential future MVMR analyses. Excluding these covariances will give the correct estimation only under the global null (β=0).

Therefore, in this section we suggest three different solutions for dealing with the lack of covariances in the GWAS summary results in order to estimate $\sigma_{km,j}$: the covariance between ${\hat{\pi }}_{k,j}$ and ${\hat{\pi }}_{m,j}$ with respect to exposure, *k*, exposure, *m* (k≠m) and SNP *j* which form the elements of $\sum_{V,j}$.

### 4.1 Estimate $\sigma_{km,j}$ from the individual level data

If some or all of the individual level data that was used in the GWAS to estimate the SNP—exposure associations is available then the covariances for the effect of each SNP on each exposure can be calculated from Equation (6).

### 4.2 Estimate the phenotypic correlation between the exposures from individual level data

The covariance for each SNP can then be approximated as;

(10) $$\sigma_{km,j}=\rho_{km}\sigma_{k,j}\sigma_{m,j},$$

where $\rho_{km}$ is the correlation between $X_{k}$ and $X_{m}$ (or phenotypic correlation). $\sigma_{kj}$ and $\sigma_{mj}$ are the SE for the effect of SNP *j* on exposures *k* and *m*, respectively. Although ideally this information would be calculated from the data used for the GWAS study, $\rho_{km}$ could also be estimated from only part of the data used in the GWAS or from an alternative dataset which is thought to have a similar structure.

### 4.3 Estimate the effect of the SNPs on each exposure from separate samples

Estimating the effect of the SNPs on each exposure in this manner means that the covariances will be zero and so excluding this information will not affect the statistics calculated. For an MVMR analysis involving *K* exposures, this would require K+1 separate samples and so is likely to only be practicable in a limited number of cases.

In any given scenario some of these solutions may be impossible (due to a lack of data) and of the solutions that are possible, one may be the most reasonable. We suggest that estimation of $\rho_{km}$ from phenotypic data, from which the appropriate covariances can then be calculated, is likely to be the most feasible and appropriate approach in many cases. Under the assumption that each SNP explains a small proportion of the variation in the exposure, the accuracy of the estimate of $\sigma_{km,j}$ will depend on the accuracy of the estimate of $\rho_{km}$. Therefore, when $\rho_{km}$ is estimated from data that does not closely match that used to estimate the SNP exposure associations exploration of how sensitive $F_{\text{TS}}$ and $\beta_{Q,\text{het}}$ are to that estimate should also be conducted. This could be done through estimation of $F_{\text{TS}}$ and $\beta_{Q,\text{het}}$ at the limits of or across the range of reasonable values of $\rho_{km}$. These results should then be used to determine whether the interpretation of the results changes over plausible values of $\rho_{km}$.

## 5 SIMULATION RESULTS

To illustrate the methods presented so far give here results from simulating and fitting MVMR models with 200 SNPs and either two or three exposures.

### 5.1 MVMR model with two exposures

**FIGURE 2:**
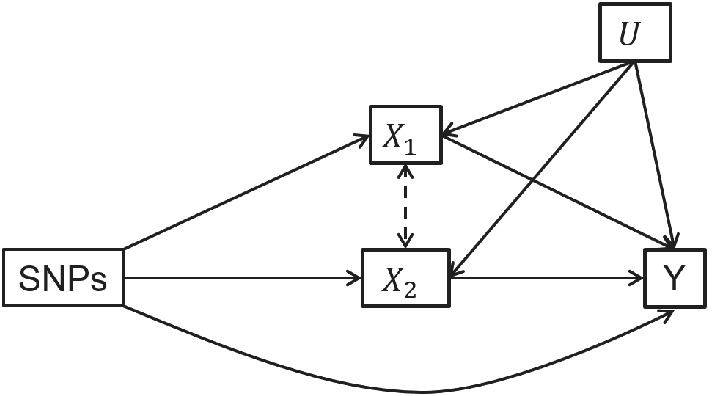
Model simulated in Table 2

First, we simulated a MVMR model with two exposures and 200 SNPs. The SNP‐exposure associations where constructed in two ways; first so that each exposure was individually and conditionally weakly predicted by the set of SNPs (ie, weak instruments) and second so that the exposures were strongly individually predicted, but weakly conditionally predicted by the set of SNPs (ie, conditionally weak instruments). In each case the association of each SNP with the exposure was drawn from a uniform distribution with the range of association selected to maintain the desired overall instrument strength. All of the SNPs were associated with both exposures, for the weak instruments there was no correlation between the association between each SNP and each exposure. Conditionally weak instruments were generated by increasing the total strength of the instruments but introducing correlation between the effect of each SNP on each of the exposures following the structure of weak instrument asymptotics first introduced by Staiger and Stock.^5^ This reflects a scenario where examination of standard *F*‐statistics for each exposure would not identify weak instruments. The exposures were simulated to both have a direct effect on the outcome and balanced pleiotropy was introduced to the model through a direct effect of the SNPs on the outcome. Pleiotropic effects were generated from a normal distribution with zero mean. A confounder of both exposures and the outcome was also included. The covariance parameter $\sigma_{i,j},\;i\neq j$ was estimated from calculation of the phenotypic correlation between $X_{1}$ and $X_{2}$ as described in Section 4. The set‐up of this model is shown in Figure 2 and results from the simulation are given in Table 2. Results for the same model without the pleiotropic effect of the SNPs on the outcome are given in Supplementary Table S1.

**TABLE 2:** Simulation results for models with heterogeneity: Two exposures, 200 SNPs

	Weak instruments		Conditionally weak instruments	
	$x_{1}$	$x_{2}$	$x_{1}$	$x_{2}$
One‐sample estimation with individual level data				
${\hat{\beta }}_{\text{OLS}}$	1.09	−0.049	0.78	−0.48
	(0.033)	(0.033)	(0.029)	(0.026)
${\hat{\beta }}_{IV}$	0.585	−0.283	0.548	−0.333
	(0.533)	(0.533)	(0.311)	(0.226)
*F*	8.80	8.80	1602.81	3107.5
	(0.61)	(0.62)	(107.67)	(208.06)
$F_{\text{SW}}$	3.40	3.40	9.75	9.78
	(0.360)	(0.360)	(0.94)	(0.95)
Two‐sample estimation with covariances				
${\hat{\beta }}_{\text{IVW}}$	0.352	−0.128	0.469	−0.276
	(0.541)	(0.541)	(0.316)	(0.228)
${\hat{\beta }}_{Q}$	$-7.7x10^{3}$	$6.7x10^{3}$	$-6.6x10^{5}$	$4.7x10^{5}$
	$\left(1.2x10^{5}\right)$	$\left(1.0x10^{5}\right)$	$\left(2.3x10^{6}\right)$	$\left(1.6x10^{6}\right)$
${\hat{\beta }}_{Q,\mathrm{het}}$	**0.487**	− **0.246**	**0.519**	− **0.313**
	**(0.777)**	**(0.778)**	**(0.350)**	**(0.253)**
$F_{\mathrm{TS}}$	**3.35**	**3.35**	**9.13**	**9.15**
	**(0.348)**	**(0.347)**	**(0.814)**	**(0.819)**
Two‐sample estimation without covariances				
${\hat{\beta }}_{\text{IVW}}$	0.352	−0.128	0.469	−0.276
	(0.541)	(0.541)	(0.316)	(0.228)
${\hat{\beta }}_{Q}$	$-6.8x10^{3}$	$6.0x10^{3}$	$-6.0x10^{5}$	$4.3x10^{5}$
	$\left(1.1x10^{5}\right)$	$\left(9.5x10^{4}\right)$	$\left(6.5x10^{5}\right)$	$\left(4.8x10^{5}\right)$
${\hat{\beta }}_{Q,\text{het}}$	0.499	−0.260	$-4.5x10^{5}$	$3.2x10^{5}$
	(0.802)	(0.803)	$\left(1.5x10^{6}\right)$	$\left(1.1x10^{6}\right)$
$F_{\text{TS}}$	3.17	3.17	0.45	0.45
	(0.337)	(0.336)	(0.054)	(0.054)

*Note*: $\beta_{1}=0.5$, $\beta_{2}=-0.3$; 4000 repetitions, 20 000 observations per repetition. Covariances estimated from the phenotypic correlation between each exposure. Weak instruments shows a scenario where the exposures are individually weakly predicted by the SNPs. Conditionally weak instruments gives a scenario where the exposures are strongly predicted by the SNPs individually but are each weakly predicted by the SNPs conditional on the other exposure.

Abbreviation: IVW, inverse variance weighted.

Results from this simulation show that the two‐sample conditional F statistic $F_{\text{TS}}$ reliably estimates the strength of the instruments and is equivalent to the conditional F statistic calculated from the individual level data $F_{\text{SW}}$ when the correlation between the exposures is used to estimate the covariance between the effect of each SNP on each exposure. These results also show that although ${\hat{\beta }}_{Q}$ does not reliably estimate the effect of the exposure on the outcome in the presence of balanced of pleiotropy, ${\hat{\beta }}_{Q,\text{het}}$ which allows for this additional heterogeneity does. This decrease in bias in ${\hat{\beta }}_{Q,\text{het}}$ compared with ${\hat{\beta }}_{\text{MVMR‐IVW}}$ when the instruments are weak comes at the cost of increased SEs, reflecting the (true) lower level of information in the model. Supplementary Table S1 shows that allowing for heterogeneity when it is not present does not increase the SE of the ${\hat{\beta }}_{Q,\text{het}}$ estimates relative to the SE of the ${\hat{\beta }}_{Q}$ estimate. The final section of Table 2 gives $F_{\text{TS}}$ and $\beta_{Q,\text{het}}$ estimated without accounting for $\sigma_{km}$. This imposes the assumption that $\sigma_{km}=0,\;\;k\neq m$, but not the assumption that $\sigma_{k}^{2}=0$ and so is a point between standard MVMR‐IVW estimation and ${\hat{\beta }}_{Q,\text{het}}$. These results also show that in the presence of conditionally weak instruments, when there is correlation between the effect of the SNPs on each exposure, if these correlations are not taken into account $F_{\text{TS},0}$ does not reliably test the strength of the instruments and ${\hat{\beta }}_{Q,\text{het},0}$ produces biased estimates of the effect of each exposure on the outcome.

### 5.2 Three exposure model

**FIGURE 3:**
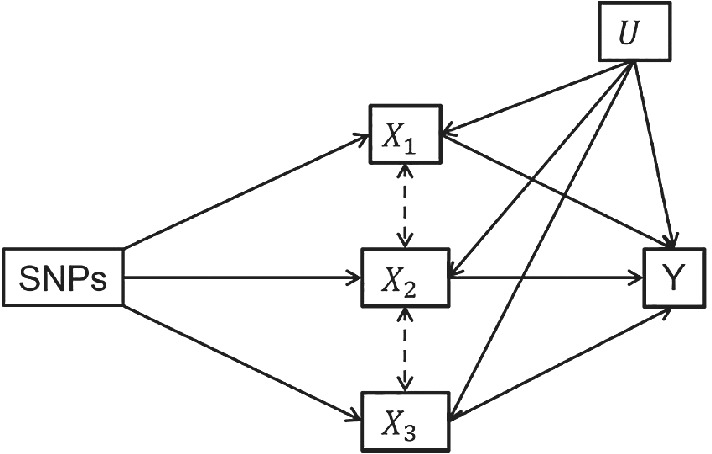
Model simulated in Table 3

Next, we simulated summary data for three exposures and 200 SNPs. Each of the exposures was simulated to have a direct effect on the outcome. All of the SNPs included in the estimation are associated with every exposure. The effect of the SNPs on the second exposure was uncorrelated with the effects on the first or third exposures. However, the effect of the SNPs on the first and third exposures were correlated, so that the third exposure was only weakly predicted by the SNPs conditional on the first exposure (and therefore the first exposure is weakly predicted conditional on the third exposure). This set up means that when only the first two exposures are included in the estimation there is directional pleiotropy present, however when all three exposures are included there is potential weak instrument bias. When the two exposures are included they each have mean conditional *F*‐statistics of 45 whereas in the model with three exposures included exposure 1 has a mean conditional *F*‐statistic of 6.5 and exposure 3 has a mean conditional *F*‐statistic 3.2. When three exposures are included in the model exposure 2 is still strongly predicted with a mean conditional *F*‐statistic of 17.9. The model under which the data was generated is shown in Figure 3 and results are given in Table 3.

**TABLE 3:** Simulation results for a model with three exposures

	Two exposures		Three exposures		
	included in estimation		included in estimation		
	$x_{1}$	$x_{2}$	$x_{1}$	$x_{2}$	$x_{3}$
One‐sample estimation with individual level data				
${\hat{\beta }}_{\text{OLS}}$	0.837	−0.065	0.667	−0.176	1.418
	(0.020)	(0.019)	(0.020)	(0.018)	(0.012)
${\hat{\beta }}_{IV}$	0.626	−0.244	0.466	−0.314	0.912
	(0.018)	(0.018)	(0.017)	(0.014)	(0.064)
*F*	236.2	235.13	236.22	235.13	14.50
	(15.43)	(13.67)	(15.43)	(13.67)	(0.89)
$F_{\text{SW}}$	78.49	78.37	6.62	19.81	3.17
	(7.10)	(6.98)	(1.13)	(5.38)	(0.29)
Two‐sample estimation with covariances				
${\hat{\beta }}_{\text{IVW}}$	0.611	−0.228	0.523	−0.277	0.502
	(0.021)	(0.021)	(0.027)	(0.021)	(0.101)
${\hat{\beta }}_{Q}$	0.626	−0.246	0.500	−0.301	0.703
	(0.022)	(0.021)	(0.034)	(0.024)	(0.149)
${\hat{\beta }}_{Q,\mathrm{het}}$	**0.624**	− **0.246**	**0.499**	− **0.301**	**0.705**
	**(0.022)**	**(0.021)**	**(0.035)**	**(0.024)**	**(0.154)**
$F_{\mathrm{TS}}$	**45.01**	**44.97**	**6.58**	**17.94**	**3.23**
	**(2.39)**	**(2.35)**	**(1.09)**	**(4.32)**	**(0.29)**

*Note*: $\beta_{1}=0.5$, $\beta_{2}=-0.3$, $\beta_{3}=0.7$; 4000 repetitions, 20 000 observations per repetition. Covariances estimated from the phenotypic correlation between each exposure. Two exposures included in estimation refers to estimation of the model including only exposures 1 and 2. Three exposures included in estimation includes exposures 1, 2, and 3.

Abbreviation: IVW, inverse variance weighted.

We give results from estimation of the model first including only two exposures, $x_{1}$ and $x_{2}$, and then including all three exposures. These results show that when only two exposures are included in the model all methods of estimating $\beta_{1}$ and $\beta_{2}$ are biased by the directional pleiotropy present in the model. When all three exposures are included in the model the MVMR‐IVW estimates are biased due to the presence of weak instruments. However, estimation of ${\hat{\beta }}_{Q}$ through minimization of $Q_{A}$ gives unbiased estimates of the effect of each exposure.

### 5.3 Heterogeneity testing

Table 4 gives the rejection rates when using $Q_{\text{IVW}}$ and $Q_{A}$ to test for pleiotropy for the model considered in Figure 2. In addition, we show rejections rates using a third heterogeneity statistic that attempts to improve $Q_{\sigma_{y}^{2}}$ by extending the weights so they take the form $\sigma_{y}^{2}+\beta^{\prime }\sum_{j}\beta$. We call this heterogeneity statistic $Q_{\text{IVW, up}}$. These extended weights are calculated using a multivariable extension of the iterative estimation described in Bowden et al.^19^ These results show that when there is no heterogeneity the null hypothesis is over rejected by both $Q_{\text{IVW}}$ and $Q_{\text{IVW, up}}$. Although the iterative updating improves on standard estimation it does not fully correct for the over rejection due to weak instruments.^19^ Estimation of $Q_{A}$ using direct minimization controls the type 1 error and when the null hypothesis is true, that is, when there is no heterogeneity this test statistic rejects approximately 5% of the time.

**TABLE 4:** Estimation of $Q_{A}$

	Weak instruments				Conditionally weak instruments			
	$\tau^{2}=0$		$\tau^{2}=0.5$		$\tau^{2}=0$		$\tau^{2}=0.5$	
	Estimate	Rej. Rate	Estimate	Rej. Rate	Estimate	Rej. Rate	Estimate	Rej. Rate
$Q_{\text{IVW}}$	228.37	41.9%	13 366.76	100%	249.71	75.4%	13 032.82	100%
	(23.04)		(678.27)		(24.85)		(770.59)
$Q_{\text{IVW, up}}$	206.20	12.9%	11 645.15	100%	201.36	7.6%	11 593.22	100%
	(21.39)		(1699.61)		(20.53)		(1338.27)
$Q_{A}$	197.16	4.5%	576.93	100%	197.74	5.2%	1788.42	100%
	(19.83)		(53.92)		(19.85)		(224.66)

*Note*: 4000 repetitions, 20 000 observations per repetition. Covariances estimated from the phenotypic correlation between each exposure.

Abbreviation: IVW, inverse variance weighted.

## 6 APPLICATION

In this section, we illustrate the use of the methods described above through an application to the estimation of the effect of multiple metabolites to AMD. AMD is disease that causes loss of central vision and is a leading cause of blindness.^22^ Elevated lipid serum levels have previously been associated with increased risk of AMD.^23^ We use data from a GWAS of 118 metabolites by Kettunen et al^16^ as our exposure and from a GWAS of AMD as our outcome.^24^ The GWAS data for our exposures included 150 SNPs that were genome‐wide signification for at least one of the metabolites. Previous studies have implicated HDL as being causal for AMD.^25^, ^26^, ^27^ In this analysis, we illustrate the issues with weak instrument bias that can arise from including multiple highly related traits in one MVMR estimation.

The GWAS data included 118 potential metabolite exposures. For the purposes of illustration we restricted the analysis to 13 metabolites moderately well predicted by a large number of SNPs. Specifically we selected the 13 metabolites that had 42 or more SNPs with an *F*‐statistic greater than 5 associated with them in our data. From the 150 SNPs included in the data we retained all SNPs which were associated with at least one of our selected exposures with an F statistic greater than 5. This gave us 78 SNPs associated with our 13 metabolites for our analysis. From this data, we considered six different models to estimate and for each one obtained the MVMR‐IVW effect estimates and investigated whether the SNPs included as instruments could conditionally predict the exposures in that model. The models considered were (a) all selected metabolites (b) to (e) subgroups of metabolites grouped by lipid fraction type and (f) a subgroup including one metabolite from each group included in (b) to (e).

Table 5 gives results for the estimation of model (a) including all of the selected metabolites. This table also reports the mean individual *F*‐statistic for the SNPs associated with each metabolite ($F_{\text{ind}}$), the mean *F*‐statistic across all of the SNPs included in the analysis for each metabolite (F) and the conditional *F*‐statistic for each metabolite ($F_{\text{TS}}$). The correlation between the metabolites, required to calculate $F_{\text{TS}}$, was not available from the GWAS data used here. We therefore calculated these using external data on the same metabolites from the Avon Longitudinal Study of Mothers and Children (ALSPAC).^28^, ^29^ A description of the ALSPAC study is given in the supplementary material. The *F*‐statistics and conditional *F*‐statistics presented for the model including all metabolites show that although each metabolite is strongly predicted by the SNPs associated with it the conditional *F*‐statistics for each exposure are very small and therefore the effect estimates are subject to weak instrument bias.

**TABLE 5:** MVMR estimates of a range of metabolites on AMD, all metabolites included in one MVMR estimation

		Estimate	SE	*P*‐value	*F*	$F_{\text{TS}}$
ApoB	ApoB	1.673	0.693	.019	10.82	0.197
IDL	IDL.PL	−4.456	0.969	<.001	11.84	0.011
	IDL.P	6.481	3.396	.061	11.76	0.626
	IDL.TG	0.437	1.391	.754	11.04	0.003
LDL	L.LDL.L	−8.695	8.376	.303	11.15	0.001
	L.LDL.P	5.223	11.125	.640	11.34	0.001
	M.LDL.P	1.794	2.360	.450	10.56	0.011
Small VLDL	S.VLDL.PL	1.054	1.530	.493	8.62	0.029
	S.VLDL.C	1.346	1.617	.408	8.88	0.005
	S.VLDL.FC	−1.270	1.331	.343	8.75	0.019
Very small VLDL	XS.VLDL.L	−6.655	1.982	.001	10.67	0.027
	XS.VLDL.P	4.866	1.668	.005	10.19	0.048
	XS.VLDL.TG	−2.384	1.819	.195	9.14	0.022

*Note: F* is the mean *F*‐statistic across all SNPs included in the estimation and is the univariable *F*‐statistic for instrument strength. $F_{\text{TS}}$ is the conditional *F*‐statistic accounting for the association between each SNP and all of the other exposures included in the estimation. 78 SNPs included in the estimation. ApoB is associated with 48 SNPs, IDL.PL, L.LDL.L, L.LDL.p, M.LDL.P, S.VLDL.PL, and XS.VLDL.L are each associated with 43 SNPs, IDL.P, IDL.TG, S.VLDL.C, S.VLDL.FC, XS.VLDL.P, and XS.VLDL.TG are each associated with 42 SNPs.

Abbreviations: AMD, age‐related macular degeneration; MVMR, multivariable Mendelian randomization; SE, standard error; SNPs, single nucleotide polymorphisms.

Table 6 gives the same results for the estimation for each subgroup of metabolites (IDL, LDL, Small VLDL, and Very small VLDL). These results show that, with the exception of IDL.PL and S.VLDL.PL, none of the metabolites are strongly conditionally predicted by the SNPs within their subgroup. For our last analysis, we included one metabolite from each group as exposures in our MVMR estimation. Table 7 gives results for this set of exposures. Although the exposures here are jointly moderately strongly predicted by the set of SNPs the conditional *F*‐statistics for each exposures are still between 4.2 and 8.3 indicating that there is likely to be some weak instrument bias. In Table 8, we re‐estimate this final MVMR model using our weak instrument robust estimators presented earlier. The results from this approach suggest that in our final model the initial MVMR‐IVW estimates may be biased towards the null due to weak instruments. $Q_{A}$ for this model is 118, the critical value at a 5% level of significance for a chi‐squared distribution with 64 degrees of freedom is 84.7. It therefore indicates potential pleiotropy and we consider the ${\hat{\beta }}_{Q,\text{het}}$ to be the most appropriate estimates in this case. Comparison of these results to those obtained from model (a) including all of the metabolites shows the potential for weak instruments to bias results of a summary‐data MVMR away from the null as well as towards the null. For three of the four metabolites included in both models the effect estimates in the final model are much closer to zero than the results in the model including all of the metabolites. The results from this analysis suggest that none of the metabolites considered are causally associated with AMD but that the standard MVMR‐IVW estimates for the final model were biased due to both weak instruments and pleiotropic effects of the SNPs on the outcome. This null result is consistent with other results using an alternative method to analyze the same data which found that HDL (not included in this analysis) was the only metabolite that was causally associated with AMD.^27^

**TABLE 6:** MVMR estimates of a range of metabolites on AMD, estimated by subgroup

	Estimate	SE	*P*‐value	*F*	$F_{\text{TS}}$
ApoB					
IDL; 54 SNPs
IDL.PL	−1.338	1.091	.226	16.05	1.23
IDL.P	1.864	1.231	.134	16.12	1.24
IDL.TG	−0.926	0.398	.024	14.97	2.23
LDL; 46 SNPs
L.LDL.L	3.707	4.341	.398	17.58	0.019
L.LDL.P	−4.781	3.484	.177	73.83	0.023
M.LDL.P	0.896	1.443	.538	16.55	0.063
Small VLDL; 50 SNPs
S.VLDL.PL	−0.513	1.021	.617	12.38	11.65
S.VLDL.C	−0.372	0.858	.667	12.42	4.75
S.VLDL.FC	0.506	1.298	.698	12.51	5.39
Very small VLDL; 53 SNPs
XS.VLDL.L	−1.651	1.863	.380	14.64	0.174
XS.VLDL.P	−0.105	0.533	.845	12.50	0.916
XS.VLDL.TG	1.395	2.112	.512	13.99	0.176

*Note: F* is the mean *F*‐statistic across all SNPs included in the estimation and is the univariable *F*‐statistic for instrument strength. $F_{TS}$ is the conditional *F*‐statistic accounting for the association between each SNP and all of the other exposures included in the estimation.

Abbreviations: AMD, age‐related macular degeneration; MVMR, multivariable Mendelian randomization; SE, standard error; SNPs, single nucleotide polymorphisms.

**TABLE 7:** MVMR‐IVW estimates of a range of metabolites on AMD including one exposure from each subgroup

	Estimate	SE	*P*‐value	*F*	$F_{\text{TS}}$
XS.VLDL.P	−0.778	0.958	.420	11.26	4.23
S.VLDL.PL	0.051	0.347	.385	9.48	5.68
L.LDL.L	0.356	0.231	.154	12.19	8.22
IDL.TG	0.067	0.761	.969	12.21	6.15

*Note*: 69 SNPs. *F* is the mean *F*‐statistic across all SNPs included in the estimation and is the univariable *F*‐statistic for instrument strength. $F_{TS}$ is the conditional *F*‐statistic accounting for the association between each SNP and all of the other exposures included in the estimation.

Abbreviations: AMD, age‐related macular degeneration; IVW, inverse variance weighted; MVMR, multivariable Mendelian randomization; SE, standard error; SNPs, single nucleotide polymorphisms.

**TABLE 8:** Weak instrument robust estimates of a range of metabolites on AMD including one exposure from each subgroup

	${\hat{\beta }}_{Q}$			${\hat{\beta }}_{Q,\text{het}}$		
	Est.	SE	*P*‐value	Est.	SE	*P*‐value
XS.VLDL.P	−5.008	3.774	.185	−2.071	1.447	.152
S.VLDL.PL	0.957	0.940	.309	0.300	0.528	.570
L.LDL.L	1.534	0.645	0.017	.728	0.613	.235
IDL.TG	2.490	2.614	0.341	.803	1.437	.576

*Note*: 69 SNPs. ${\hat{\beta }}_{Q}$ gives the estimate obtained by minimization of *Q*, ${\hat{\beta }}_{Q,\text{het}}$ gives the estimate obtained by minimization of *Q* allowing for balanced pleiotropy.

Abbreviations: AMD, age‐related macular degeneration; SE, standard error; SNPs, single nucleotide polymorphisms.

## 7 SOFTWARE

We have written an R package MVMR which facilitates the implementation of MVMR estimation and corresponding sensitivity analyses. The package requires summary data on instrument‐exposure and instrument‐outcome associations, as well as information on the pairwise covariances of the error in the estimated association between each SNP and each pair of exposures. As these covariances are often not available the software can be implemented in three ways; estimating the covariances from individual level data, approximating the covariances from the phenotypic correlation between the exposures or assuming that these covariances are zero.

### 7.1 Workflow

Fitting and interpreting MVMR using the methods described in this article, including tests for instrument strength and horizontal pleiotropy, is performed using a five‐step procedure. Initially, summary data should be provided, including a covariance matrix for the effect of the genetic variants on each exposure. As such covariances are not conventionally reported in publicly available data, two functions snpcov_mvmr() and phenocov_mvmr() can be used to generate the covariance matrix. The function snpcov_mvmr() estimates the covariance terms directly from individual level data, whilst phenocov_mvmr() uses the phenotypic correlation and summary data (input by the user) to generate estimates of the covariances.

**FIGURE 4:**
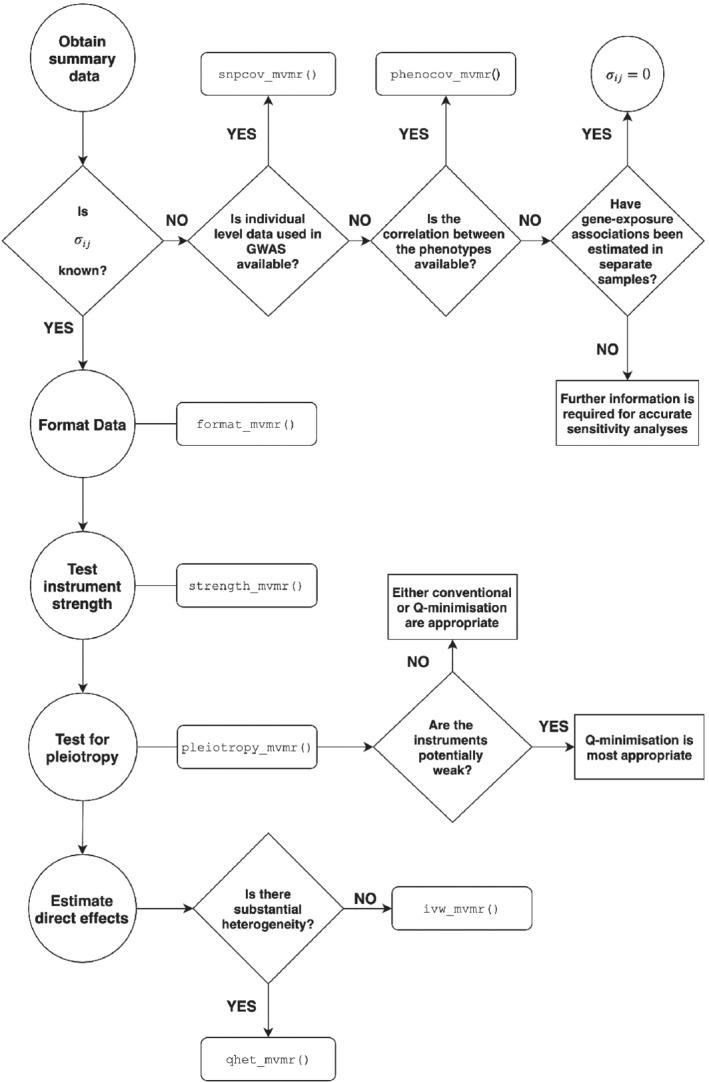
Workflow for multivariable Mendelian randomization R package

As a second stage, the summary data is reformatted using the function format_mvmr() into a data frame which is subsequently used as the input for estimation and sensitivity analyses. We then provide the functions strength_mvmr() to evaluate instrument strength using the two sample conditional *F*‐statistic described in Section 2. Tests for horizontal pleiotropy are performed using pleiotropy_mvmr(), performing both standard and *Q*‐minimization approaches simultaneously (see Section 3 for more details). Finally, causal effects can be estimated using two different approaches; fitting an IVW MVMR model using ivw_mvmr() and minimizing the *Q*‐statistic allowing for heterogeneity using qhet_mvmr(). Each step in the MVMR workflow is shown in Figure 4. The MVMR package is available to download at https://github.com/WSpiller/MVMR/. The package also includes a detailed tutorial demonstrating functionality of the package in an analyses of the effects of lipid fractions upon systolic blood pressure using data from the Global Lipids Genetics Consortium and UK Biobank.

## 8 DISCUSSION

In this article, we develop a general statistical framework for conducting two sample MVMR analyses for an arbitrary number of exposures in the presence of weak instrument bias and pleiotropy. The methods presented here give ways to test for weak instruments in two‐sample MVMR and to robustly test for heterogeneity due to pleiotropy in the presence of moderately weak instruments. We additionally give a method to estimate causal effects in the presence of moderately weak instruments which is robust to balanced pleiotropy.

Weak instruments are a potential issue in many applications where estimating direct effects of multiple exposures using MVMR is preferred over univariable MR analyses, which are thought to be likely to be affected by directional pleiotropy.^30^, ^31^, ^32^, ^33^, ^34^ MVMR approaches are also used to gague the extent to which one exposure mediates the effect of another on the outcome.^35^, ^36^ Any application of MVMR will be biased by conditionally weak instruments and, as illustrated by our application, this can occur even when the genetic variants strongly predict each exposure individually. Therefore, the methods presented here are important as they provide a way to identify and correct for weak instruments in two‐sample MVMR estimation.

The $F_{\text{TS}}$ statistic described here is calculated using estimates of $\hat{\delta }$ calculated from an IVW estimation of the effect of ${\hat{\pi }}_{-k}$ on ${\hat{\pi }}_{k}$. An alternative method of estimation, equivalent to that described for estimation of β, is to directly minimize its constituent $Q_{x_{k}}$ to obtain LIML estimates for δ.^19^, ^20^ Whilst this procedure enacted on the $Q_{A}$ statistic furnishes attractive, weak instrument robust causal estimates, initial simulation results (not reported here) showed limited benefit of estimating δ in this way therefore we did not investigate potential implementation further.

There are a number of limitations to this work. The test statistic and weak instrument robust estimation requires an estimate of the covariance between the error in the estimated effect of each SNP on each exposure. Our simulation results highlight how important this data can be as the estimated values of $F_{\text{TS}}$ and ${\hat{\beta }}_{Q,\text{het}}$ are changed so they become uninterpretable when this covariance is fixed to zero. Although this data is generally not available we propose a method to estimate it, using the phenotypic correlation between the exposures, which can be used to obtain a reasonable approximation if the relevant covariance when each SNP only explains a small proportion of each exposure. Where the data used to estimate the correlation between the exposures is the same data used to estimate the SNP‐exposure associations the estimated value of $\sum_{V,j}$ will closely match the true value. When this is not the case the level of error in $F_{\text{TS}}$ and $\beta_{Q,\text{het}}$ that results from misspecification of $\rho_{km}$ will depend on the other parameters in the model. For $F_{\text{TS}}$ this will depend on how related the exposures are and how strongly (or weakly) they are predicted. Misspecification of the conditional *F*‐statistic will not matter if it is notably larger (or smaller) than 10 for all possible values of $\sum_{V,j}$ as this will not change the interpretation of the results. For $\beta_{Q,\text{het}}$ how much the specification of $\sum_{V,j}$ matters will depend on the estimated effect of each exposure, if all or all but one of these are zero $\sum_{V,j}$ will not affect the estimated results, and the magnitude of any effect will depend on the size of these estimated effects. When the data used for the estimation of $\rho_{km}$ does not match that used to obtain the SNP‐exposure associations we have therefore proposed that the researcher investigates how variation in $\rho_{km}$ affects the obtained values, and interpretation of $F_{\text{TS}}$ and ${\hat{\beta }}_{Q,\text{het}}$. Where plausible variation in $\rho_{km}$ does affect the interpretation of the results this limitation, and the resulting potential interpretations of the results obtained, should be accounted for by the researcher applying this method.

Another weakness of the test statistics provided here is the lack of SEs for the point estimates of the direct effect of each exposure. We propose using a jackknife to estimate these SEs. This does however make the estimation of these statistic more computationally intensive than would the case if the SEs could be calculated analytically.

The weak instrument robust point estimates are robust to weak instruments but cannot produce reliable estimates when instruments become very weak or if only a small number of SNPs are available. Although, we show this method works with moderately weak instruments it is not clear exactly how weak is too weak, or indeed how few instruments are too few, to produce either reliable point estimates or heterogeneity statistics. Gaining a more precise understanding of these questions is a topic for further research.

Although, we propose weak instrument robust estimation, if the weak instruments are limited to only a small number of the exposures in the model an alternative approach may be to drop exposures (one at a time) until the conditional *F*‐statistics show that all of the exposures are strongly predicted by the SNPs. This would however need to be considered carefully by the researcher. The model to be estimated should not be decided purely by which exposures can be predicted but driven by a research question of interest and dropping exposures has the potential to introduce directional pleiotropy into the estimation biasing the resulting effect estimates. The choice of approach to take would depend on the number of SNPs and exposures in the estimation and the relationship between the exposures as well as how weak the SNPs are as instruments. As illustrated by our application these approaches could be combined, excluding exposures until instrument strength is high enough to reasonably apply the weak instrument robust methods. The choice of approach needs to be considered on a case by case basis.

Additionally although our final estimation $\beta_{Q,\text{het}}$ is robust to balanced pleiotropy it will still give biased estimates in the presence of unbalanced or directional pleiotropy. Multivariable MR Egger,^14^ has been proposed as a method for obtaining reliable MVMR estimates in the presence of directional pleiotropy. Extending this approach to account for weak instrument bias is another topic of further research.

Box 1: **Summary of statistics discussed in this article.**

**Instrument strength statistics;**

*F*—Measure of the strength of the instruments to predict one exposure. Applies to individual or summary level data and to univariable or multivariable MR estimation.

*Conditional F‐statistic* $F_{\text{SW}}$—Measure of the strength of instruments to predict one exposure conditional on the other exposures included in the estimation. Applies to multivariable MR estimation with individual level data.

*Conditional F‐statistic* $F_{\text{TS}}$—Measure of the strength of instruments to predict one exposure conditional on the other exposures included in the estimation. Applies to multivariable MR estimation with summary data.

$Q_{x_{j}}$—A *Q*‐statistic from which $F_{\text{TS}}$ is calculated.

**Heterogeneity statistics;**

$Q_{\text{IVW}}$—A heterogeneity test for MVMR that uses the IVW point estimates and does not account for the uncertainty in the estimated SNP‐exposure associations. This test over rejects the null in the presence of weak instruments.

$Q_{\text{IVW, up}}$—A heterogeneity test for MVMR that uses the IVW point estimates but accounts for the uncertainty in the estimated SNP‐exposure associations. This test over rejects the null in the presence of weak instruments, but to a lesser extent that $Q_{\text{IVW}}$.

$Q_{A}$—A heterogeneity test for MVMR that is robust to weak instruments, in the sense that it has the appropriate type 1 error rate in the presence of weak instruments.

**Estimation statistics;**

${\hat{\beta }}_{\text{IVW}}$—Estimates of the causal effect of each exposure on the outcome, estimated using standard IVW.

${\hat{\beta }}_{Q}$—Estimates of the causal effect of each exposure on the outcome, estimated through minimization of $Q_{A}$. Robust to weak instruments.

${\hat{\beta }}_{Q,\text{het}}$—Estimates of the causal effect of each exposure on the outcome, estimated through minimization of $Q_{A}$ with an additional parameter to account for heterogeneity. Robust to weak instruments and pleiotropy.

Box 2: **Recommended tests in Two‐sample MVMR.**

In all two‐sample summary data MVMR estimation two statistics should be calculated;

1. Conditional F statistics, $F_{\text{TS}}$, for each exposure.
  These test the strength of the genetic variants to predict each exposure in the multivariable mode. $F_{\text{TS}}<10$ suggests potential weak instrument bias in the MVMR estimation.
2. A *Q*‐statistic for heterogeneity, $Q_{A}$, for the model.
  Rejection of $Q_{A}$ using standard significant levels (eg, p<0.05) indicates potential pleiotropy in the form of excessive heterogeneity in the MVMR model. However, this test will often reject in the presence of weak instruments.
  If weak instruments are detected, that is, any of the $F_{\text{TS}}$ values are less than 10, IVW‐MVMR estimates are potentially biased. When large numbers of SNPs are available this can be corrected through;
3. Estimating ${\hat{\beta }}_{Q,\text{het}}$ for each exposure
  This method gives estimates of the direct effect of each exposure on the outcome that are robust to (moderately) weak instruments.
4. An updated $Q_{A,\min}$ which minimizes the *Q* statistic over $\beta_{Q}$.
  This test provides a test for heterogeneity that has the correct size in the presence of weak instruments. Rejection of $Q_{A,\min}$ using standard significant levels (eg, p<0.05) indicates potential pleiotropy in the MVMR model even in the presence of moderately weak instruments.

All of these tests and estimation statistics are provided in the MVMR R package.

## AUTHOR CONTRIBUTIONS

E.S. and J.B. devised the project. E.S. conducted the analysis and wrote the first draft of the paper. W.S. developed the software package. All authors reviewed and approved the final version.

## CODE AVAILABILITY

The code used to conduct the simulations and applied analysis is available at https://github.com/eleanorsanderson/MVMRweakinstruments. The MVMR package is available at https://github.com/WSpiller/MVMR/.

## Supporting information

Appendix S1

## Data Availability

Details and data for the data used in this article are available at https://github.com/WSpiller/MRChallenge2019. We additionally use some data from ALSPAC, details of the ALSPAC cohort and data access are available at http://www.bristol.ac.uk/alspac.
